# Hemopexin alleviates cognitive dysfunction after focal cerebral ischemia-reperfusion injury in rats

**DOI:** 10.1186/s12871-019-0681-2

**Published:** 2019-01-15

**Authors:** Beibei Dong, Yongyan Yang, Zhishen Zhang, Keliang Xie, Lin Su, Yonghao Yu

**Affiliations:** 1 Department of Anesthesiology, Tianjin Institute of Anesthesiology, General Hospital of Tianjin Medical University, No. 154 Anshan Road, Heping District, Tianjin, 300052 People’s Republic of China; 2 Department of Anesthesiology, Xiamen Medical University Zhongshan Hospital, Xiamen, 516211 China

**Keywords:** Hemopexin, Heme-oxygenase-1, Cerebral ischemia-reperfusion injury, Blood-brain barrier

## Abstract

**Background:**

Ischemia-reperfusion (I/R) is a critical pathophysiological basis of cognitive dysfunction caused by ischemia stroke. Heme-oxygenase-1 (HO-1) is the rate-limiting enzyme for the elimination of excessive free heme by combining with hemopexin (HPX), a plasma protein that contributes to eliminating excessive free heme during ischemia stroke. This study aimed to elucidate whether HPX could alleviate cognitive dysfunction in rats subjected to cerebral I/R.

**Methods:**

Rats were randomly divided into five groups: sham, MCAO, Vehicle, HPX and HPX + protoporphyrin IX (ZnPPIX). Cerebral I/R was induced by MCAO. Saline, vehicle, HPX and HPX + ZnPPIX were injected intracerebroventricularly at the moment after reperfusion. Morris water maze (MWM) test was used to detect the learning and cognitive function. Western blot was used to detect the expression of HO-1 in ischemic penumbra. CD31/vWF double labeling immunofluorescence was used to detect the neovascularization in the penumbra hippocampus. The structure and function of blood-brain barrier (BBB) was detected by the permeability of Evans Blue (EB), water content of the brain tissue, the Ang1/Ang2 and VE-cadherin expression.

**Results:**

Our study verified that HPX improved the learning and memory capacity. Hemopexin up-regulated HO-1 protein expression, the average vessel density in the penumbra hippocampus and the VE- cadherin expression but decreased the permeability of EB, the water content of brain tissue and the ratio of Ang1/Ang2. The effects were reversed by ZnPPIX, an inhibitor of HO-1.

**Conclusion:**

HPX can maintain the integrity of the blood-brain barrier and alleviate cognitive dysfunction after cerebral I/R through the HO-1 pathway.

## Background

Cognitive dysfunction is the main cause of the decline in the quality of life in patients subjected to stroke, which is well known for its high incidence, disability, mortality and recurrence rate worldwide [1]. However, there is no effective and efficient therapeutic strategy in clinical practice because of its complicated and still unclear pathological mechanism and narrow therapeutic time window. For this reason, the further exploration of its underlying signaling mechanism and the investigation of novel promising interventions to alleviate the cognitive dysfunction resulting from stroke are necessities.

Emerging evidence has shown that ischemia-reperfusion (I/R) injury plays a significant role in the development of stroke [2]. When I/R injury occurs, endothelial injury and dysfunction leads to the blood-brain barrier damage, which in turn, aggravates I/R injury [3]. Additionally, the free heme released from methemoglobin during cerebral I/R is a toxic component of endothelial cells [4]. In the CNS, free heme was shown to be toxic to both cortical neurons and astrocytes in established in vitro and in vivo models [5]. Hemopexin (HPX) is a 60-kDa serum glycoprotein that is synthesized locally by cells of the central nervous system. HPX exhibits the greatest binding affinity (*K*_*d*_ < 1 pM*)* for heme and, thus, behaves as an efficient scavenger of overloaded toxic heme.

Our previous study showed that hemopexin expression was increased in neurons and astrocytes in the penumbra area 24 h after ischemia-reperfusion. Intracerebroventricular injection of HPX reduced the infarct volumes and improved measurements of neurological function within 7 d after MCAO. The neuroprotective effects of HPX were sustained for 7 d after ischemia-reperfusion [6]. Heme oxygenase 1 (HO-1) is the rate-limiting enzyme in the degradation of free heme [7]. Emerging evidence has shown that HO-1 play an important role in protecting the blood brain barrier of cerebral infarction [8]. Furthermore, HO-1 can up-regulate the number of circulating circulating endothelial progenitor cells (EPCs) and to alleviate the multiple organ injury induced by ischemia-reperfusion injury [9]. In the present study, we designed experiments to explore whether HPX could improve cognitive dysfunction associated with cerebral ischemia-reperfusion injury, and to determine whether this effect is associated with HO-1.

## Methods

### Ethics statement and animal preparation

All protocols carried out in this article were approved by the Medical University of Tianjin experimental animal management committee (Aecl2015–0158 [JIN]; October 27, 2015). Male Sprague–Dawley (SD) rats (7 to 8 weeks old, weighting 250 g to 280 g) provided by Experimental Animal Laboratories of the Academy of Military Medical Sciences (license number: SCXK_ (Army) 2009–003, Beijing, China), were housed individually with a 12-h light/dark cycle, relative humidity of 55 to 75% and a constant surrounding temperature (22 ± 2 °C), with water and food available ad libitum. All rats were randomized into treatment groups (Sham *n* = 36; MCAO *n* = 36; Vehicle *n* = 36; HPX *n* = 36; HPX + ZnPPIX *n* = 30), and researchers were blinded to treatments in all experiments below. Rats in the sham group received a sham operation; in the other groups, rats all received MCAO with or without intracerebroventricular injection of HPX (HPX group) and HPX with ZnPPIX (HPX + ZnPPIX group). Euthanasia was performed by intraperitoneal injectition of pentobarbital sodium (200 mg/kg) after the experiments.

### Surgical procedure

The middle cerebral artery occlusion (MCAO) method was applied to induce the focal cerebral ischemia/reperfusion (I/R) injury model. Briefly, rats were fasted for 12 h before surgery with water accessible and were then anesthetized with chloral hydrate (10%, 3 ml/kg) by intraperitoneal injection. Then, a ventral midline neck incision was made, followed by the identification and careful free dissection of the right common carotid artery (CCA), external carotid artery (ECA), and internal carotid artery (ICA) from surrounding tissues. A surgical filament (Beijing Cinontech Co. Ltd.) was inserted into the ICA after the proximal ligation of the CCA and ECA until a mild resistance was felt, indicating the occlusion of the middle cerebral artery (MCA), which resulted in a transient cessation of blood flow and subsequent brain infarction in the area supplied by the MCA. The skin was closed with a 4–0 silk suture, followed by 1 h of ischemia. After that, the filament was withdrawn to initiate a reperfusion process. The body temperature of the rats was maintained at 37 ± 0.5 °C during the whole procedure.

### Intracerebroventricular injection

Right after reperfusion, rats were anesthetized as before and placed on a stereotaxic frame. The skull was fixed and secured with four stainless-steel screws, and a scalp incision was performed to expose the surface of the skull and bregma. A burr hole (1.5 mm lateral to and 0.8 mm posterior to bregma) was drilled into the right cranial bone. A 25-μl microsyringe (Hamilton) was very slowly inserted 3.5 mm beneath the dural surface to inject saline (10 μl, 0.9% sodium chloride for the MCAO group rats), vehicle (10 μl, 0.1% sodium azide for the vehicle group rats), rat hemopexin reference serum (10 μl, 1.86 g/l HPX dissolved in 0.1% sodium azide, ICL, Portland for the HPX group rats) or solution mix (10 μl, HPX 1.86 g/l + ZnPPIX 20 μM for the HPX + ZnPPIX group rats) once at a rate of approximately 3.3 μl·min^− 1^ within 3 min with a semi-automatic control device. The microsyringe was kept in place for 5 min to ensure the effectiveness of injection before withdrawal. After withdrawal, the incision was sutured, and the rats were allowed to recover from anesthesia. Sham rats underwent the same surgical procedure without exposure to MCAO injury and cerebroventricular injection.

### Morris water maze (MWM) assay

The MWM assay consists of two parts: a spatial probe test and a hidden platform test [10]. Briefly, the test system included a large four-quadrant circular tank (210 cm in diameter and 50 cm in height) filled with water (temperature was maintained at 19 to 22 °C, 30 cm in depth) and colored cards on the walls that remained constantly throughout the whole experiment. In the spatial test, a black target platform was fixed 1 cm below the surface of the water in the middle of the southwest (SW) quadrant, which ensured that the platform was invisible to rats. Each rat was tested four times a day, with a 5-min interval between each trial, for 5 consecutive days and randomly released in quadrant positions as described below. Each trial had a maximum duration of 120 s, and rats were allowed to swim freely in the tank to find the hidden platform. Once the hidden platform was found, the rats were allowed to rest on the platform for 30 min, and the time spent to find the platform was recorded as their escape latency. If rats failed to find the platform within 120 s, they were placed on the platform for 30 s by the researcher, and the escape latency was recorded as 120 s. After the 5 days of tests, the hidden platform test was carried out. In this test, the hidden platform described above was removed to observe and record the time spent (time percentage) in the target quadrant (quadrant SW) and the number of platform crossings. The escape latency, time percentage and number of platform crossings in the MWM were recorded by a computerized video tracking system (Ethovision 3.0; Noldus Information Technology, Wageningen, Netherlands).

### Western blot analysis

At 7 days after ischemia-reperfusion, rats were sacrificed to quickly obtain the brain tissue of the ischemic penumbra, which was snap-frozen in liquid nitrogen. The ischemic penumbra brain tissue was then ground in radioimmunoprecipitation (RIPA) assay buffer (Sigma-Aldrich) containing protease inhibitors to obtain the protein extracts. An equal amount (40 μg) of protein sample among the groups was loaded into each lane of a 10% sodium dodecyl sulfate (SDS) polyacrylamide gel and separated by electrophoresis, followed by electrotransference to a polyvinylidene difluoride (PVDF) membrane (Millipore, Billerica, MA). After being blocked with 5% nonfat milk for 2 h at room temperature, membranes were incubated with the following primary antibodies: anti-HO-1 (rabbit polyclonal, 1:1000 in dilution, Santa Cruz Biotechnology Inc., USA), anti-VE-cadherin (rabbit polyclonal,1:3000 in dilution, abcam, USA) and anti-rabbit β-actin (rabbit polyclonal, 1:1000 in dilution, Abcam, USA) with gentle shaking at 4 °C overnight. The membranes were washed in TBST (Tris-buffered saline Tween-20) 3 times (5 min each time), followed by incubation with the corresponding goat anti-rabbit secondary antibodies (11,000 dilution, CWBIO, Peking, China) to recognize the bound antibodies at room temperature for 2 h. The immunoblots were immersed in enhanced chemiluminescent (ECL) reagent to visualize the specific protein bands, followed by exposure to ECL-hyperfilm (Amersham Biosciences, Piscataway, NJ, USA).

### Immunofluorescence staining

Rats were sacrificed after being intraperitoneally anesthetized at 7 days after reperfusion. The rats were anesthetized intraperitoneally as described before and transcardially perfused with heparinized saline, followed by transcardial perfusion with 4% paraformaldehyde. After perfusion, the rats were decapitated to remove the brain tissue from the skull, embed it within OCT medium (Leica 0201 06926, Germany) and store it at − 80 °C. Coronal sections (8 μm) were obtained from the frozen brain tissue for immunohistochemical staining. CD31 and vWF are two endothelial cell markers. After deparaffinization and dehydration, non-specific endogenous peroxidase activity was blocked by treating sections with 3% hydrogen peroxide in methanol for 30 min. Antigen recovery was performed by boiling the sections for 10 min in 10 mM citrate buffer (pH 6.0). Nonspecific binding was blocked with 1% non-immune serum in PBS for 30 min. The sections were then incubated with a goat monoclonal antibody (mAb) against CD31 (1:100, Abcam, England) and vWF (1:100, Abcam, England) overnight at 4 °C. The sections were then washed with PBS, incubated with a biotinylated anti-goat IgG (1:100, Santa Cruz Biotechnology) for 2 h at 37 °C, washed and then incubated with an avidin peroxidase conjugate solution (1:100, Santa Cruz Biotechnology) for 1 h. Finally, the sections were developed with diaminobenzidine for 5 min. Negative controls were similarly processed but without the primary antibody.

### CD31 and vWF measurements

The vascular density and the number of CD31-positive cells in the injured brain were measured by vWF and CD31 immunofluorescence staining. Here, five brain tissue sections at 50-mm intervals were analyzed under a light microscope (200×, Leica). The vWF-positive vessels in the boundary zone of the lesion and in the injured hippocampus were counted. The vascular density and the number of CD31-positive cells in both regions were determined by dividing the immuno-reactive vessels by the corresponding area [11]. Data presented as total number of vessels or CD31 per mm.

### Permeability of brain blood barrier

The BBB permeability was evaluated by Evans blue (EB) dye extravasation. Two days after MCAO, EB (2.5%, 2 ml/kg) was injected through the femoral vein 1 h before euthanasia. The brains were harvested after perfusion with 4% paraformaldehyde, and were then incubated in dimethylformamide at 60 °C for 24 h. Then the brain tissue was homogenized and centrifuged at 1000 rpm for 5 min. Standard curve was drawn by testing the absorbance of gradient-dosed Evans blue. The absorbance of the supernatant was detected at 625 nm to calculate the relative content of EB. Brain water content was measured by the dry-wet method as previously reported and was expressed as a percentage of wet weight [12].

### Quantitative real-time polymerase chain reaction

For quantitative real-time polymerase chain reaction (qRT-PCR), Total RNA was extracted from the frozen brain tissue described above with Trizol reagents (Roche, Germany). In addition, the concentration of total RNA was measured by an OD of 260 nm on a Smart Spec™ plus spectrophotometer (Bio-Rad, Hercules, CA). RNA was reversely transcribed to complementary DNA (cDNA) using a Transcriptor First Strand cDNA Synthesis Kit (Roche, Germany). qRT-PCR was performed according to the instructions of the manufacture’s amplification kit (Roche, Germany), using the SYBR Green method on an ABI 7500 instrument (Applied Biosystems, Foster City, CA). The RT-PCR amplification was performed with a volume of 50 μl using a ready-to-use qRT-PCR mix (Roche, Germany). All reactions were performed in triplicate. The messenger RNA (mRNA) levels were normalized to the endogenous reference gene GAPDH. Relative quantification was achieved by the comparative 2^−ΔΔct^ method as described [13]. The sequences of the primers used for RT-PCR are shown below (Tab. 1).

### Statistical analysis

Data were analyzed using SPSS 15.0 (SPSS Inc., Chicago, IL). All data were expressed as the mean ± SD. A one-way analysis of variance (ANOVA) with a Tukey post hoc test was used to compare mNSS, MWM and the vascular density in the brain tissue between the three different groups. Two-way ANOVA was used for escape latency. *P* value of 0.05 was considered to be statistically significant.

## Results

### HPX improved the long-term spatial learning and memory ability in rats after focal cerebral I/R injury

**Fig. 1:**
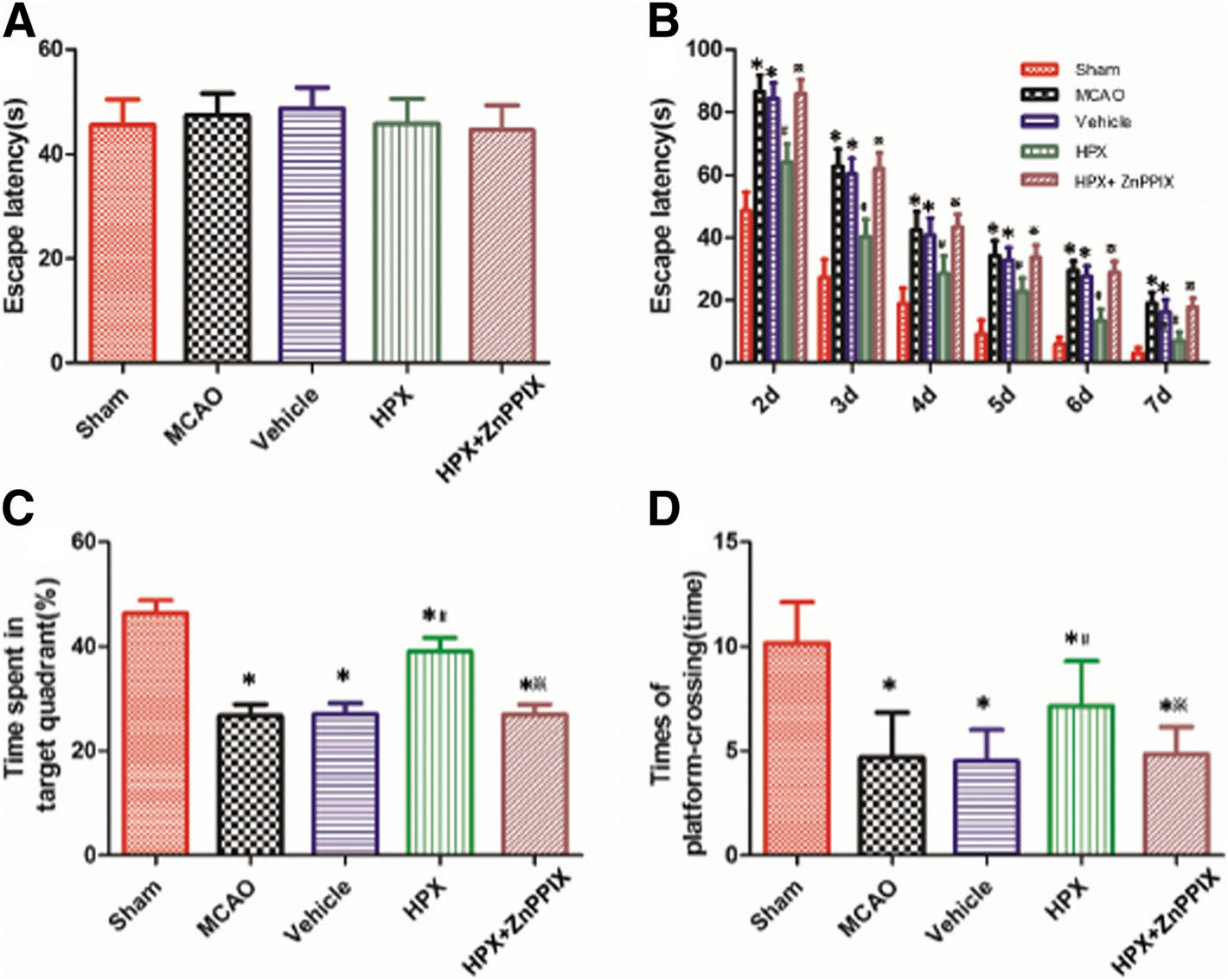
Intracerebroventricular HPX injection improved the long-term spatial learning and memory ability in rats after focal I/R injury. The baseline escape latencies in the spatial probe test among the five groups before sham operation and focal cerebral I/R injury (− 24 h) were not significantly different (**a**). Compared with rats in the sham group, rats in the MCAO group needed more time to find the platform hidden under water in the spatial probe test (**b**) at 2–7 d after focal I/R injury, spent less time in the target quadrant (**c**) and crossed the platform fewer times in the hidden platform test (**d**) at 7 d after focal I/R. Compared with the rats in the vehicle group, the rats in the HPX group exhibited a shorter escape latency on day 2 to 7 after focal cerebral I/R injury in the spatial probe test (**b**) and a increase in the time spent in the target quadrant (**c**) and number of platform crossings (**d**) in the hidden platform test at 7 d after focal I/R. The inhibitor of HO-1, ZnPPIX, blocked the effect of HPX on shortening the escape latency in the spatial probe test and increasing the time spent in the target quadrant (**c**) and number of platform crossings (**d**) in the hidden platform test. However, there were no significant differences in the escape latency in the spatial probe test (**b**), the time spent in the target quadrant (**c**) or the number of platform crossings (**d**) in the hidden platform test between the MCAO and vehicle group after focal cerebral I/R injury. ^*^P < 0.05 vs. sham group; ^#^*P* < 0.05 vs. vehicle group; ^※^*P* < 0.05 vs. HPX group

The baseline escape latency in the spatial probe test among the five groups before sham operation and focal cerebral I/R injury (− 24 h) was not significantly different (*P* > 0.05, *n* = 6, Fig. 1a). Compared with the escape latency of the sham group, the escape latency of the MCAO group in the spatial probe test on day 2 to 7 after focal cerebral I/R injury was significantly longer (Sham vs. MCAO: 48.58 ± 5.99 s vs. 86.56 ± 5.24 s, 27.23 ± 5.82 s vs. 62.76 ± 5.53 s, 18.76 ± 5.14 s vs. 42.39 ± 5.91 s, 9.10 ± 4.41 s vs. 34.09 ± 4.89 s, 6.03 ± 2.18 s vs. 29.47 ± 3.05 s, 3.11 ± 1.67 s vs. 18.96 ± 3.55 s; *P* < 0.05, *n* = 6, Fig. 1b), and the time spent in the target quadrant and number of platform crossings in the hidden platform test were dramatically lower in the MCAO group than in the sham group (Sham vs. MCAO: 46.29 ± 2.51 s vs. 26.66 ± 2.32 s and 10.17 ± 1.94 vs. 4.67 ± 2.16; *P* < 0.05, *n* = 6, Fig. 1c and d). In the HPX group, compared with the measures in the vehicle group, the escape latencies in the spatial probe test on day 2 to 7 after focal cerebral I/R injury were significantly lower (Vehicle vs. HPX: 84.32 ± 5.01 s vs. 64.01 ± 5.98 s, 60.37 ± 5.01 s vs. 40.22 ± 5.62 s, 40.72 ± 5.59 s vs. 28.61 ± 5.55 s, 32.67 ± 4.22 s vs. 22.80 ± 4.12 s, 27.53 ± 3.44 s vs. 13.34 ± 3.78 s, 16.32 ± 3.79 s vs. 6.87 ± 3.03 s; *P* < 0.05, *n* = 6, Fig. 1b), and the time spent in the target quadrant and number of platform crossings in the hidden platform test were dramatically increased (Vehicle vs. HPX: 26.96 ± 2.13 s vs. 39.00 ± 2.69 s and 4.50 ± 1.52 vs. 7.17 ± 2.14, respectively; *P* < 0.05, *n* = 6, Fig. 1c and d). The escape latencies in the spatial probe test on day 2 to 7 after focal cerebral I/R injury in the HPX + ZnPPIX group were obviously longer than those in the HPX group (HPX vs. HPX + ZnPPIX: 64.01 ± 5.98 s vs. 85.76 ± 4.51 s, 40.22 ± 5.62 s vs. 62.03 ± 5.01 s, 28.61 ± 5.55 s vs. 43.44 ± 4.03 s, 22.80 ± 4.12 s vs. 33.65 ± 3.98 s, 13.34 ± 3.78 s vs. 28.87 ± 3.47 s, 6.87 ± 3.03 s vs. 17.77 ± 2.89 s; *P* < 0.05, *n* = 6, Fig. 1b), and the time spent in the target quadrant and number of platform crossings in the hidden platform test were both dramatically decreased in the HPX + ZnPPIX group (HPX vs. HPX + ZnPPIX: 39.00 ± 2.69 s vs. 26.86 ± 2.13 s and 7.17 ± 2.14 vs. 4.83 ± 1.33, respectively; *P* < 0.05, *n* = 6, Fig. 1c and d). However, there were no significant differences in the escape latency, time spent in target quadrant and number of platform crossings between the MCAO and vehicle group (MCAO vs. Vehicle: *P* > 0.05, *n* = 6, Fig. 1b, c and d).

### HPX treatment up-regulated HO-1 expression in the cerebral ischemic penumbra area

**Fig. 2:**
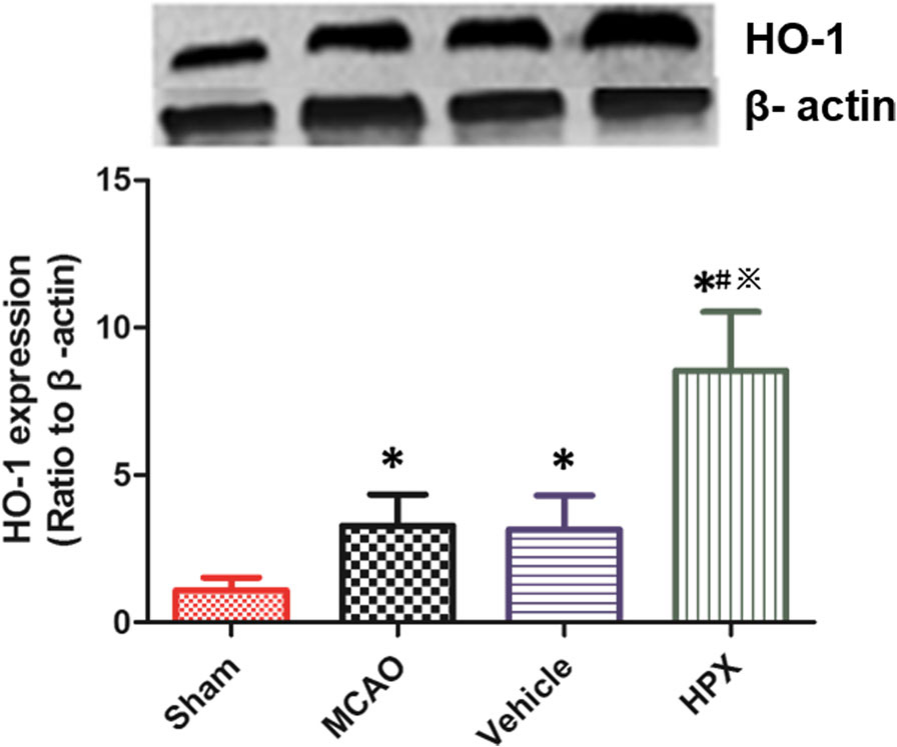
HPX up-regulated the expression of HO-1 on day 7 after focal cerebral I/R injury. When compared with the rats in the sham group, those with focal cerebral I/R injury exhibited an increase in the HO-1 protein expression on day 7 after I/R injury. The HO-1 protein expression was increased in the vehicle and MCAO group. HPX injection up-regulated the HO-1 expression. ^*^P < 0.05 vs. sham group; ^#^*P* < 0.05 vs. MCAO group; ^※^*P* < 0.05 vs. vehicle group

Compared with the sham group, the MCAO group exhibited an increase in HO-1 protein expression (Sham vs. MCAO: 2.16 ± 0.86 vs. 5.33 ± 1.45, *P* < 0.05, *n* = 6, Fig. 2) in the ischemic penumbra on day 7 after focal cerebral I/R injury. In the HPX group, compared with the protein expression of HO-1 in the MCAO group and vehicle group, the protein expression of HO-1 (MCAO vs. HPX: 3.28 ± 1.07 vs. 8.53 ± 2.01, *P* < 0.05; Vehicle vs. HPX: 3.16 ± 1.15 vs. 8.53 ± 2.01, *P* < 0.05, *n* = 6, Fig. 2) were markedly higher. However, there were no significant differences between the MCAO and vehicle group in the protein expression levels of HO-1 (MCAO vs. Vehicle: *P* > 0.05, *n* = 6, Fig. 2).

### HPX improved the neovascularization of the ischemic penumbra hippocampus in rats after focal cerebral I/R injury

**Fig. 3:**
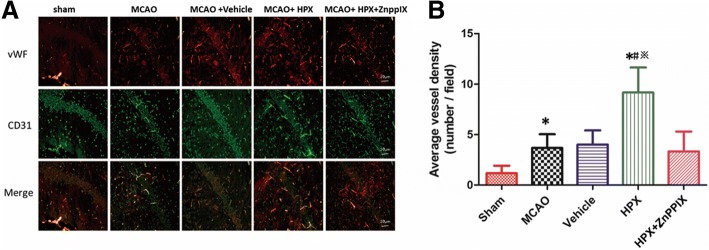
Intracerebroventricular HPX injection enhanced the neovascularization of the ischemic penumbra hippocampus in rats after focal cerebral I/R injury. Compared with the respective densities in the sham group, the new vessel densities in the ischemic penumbra hippocampus (**a**) in rats of the MCAO group were higher at 7 days after focal cerebral I/R injury. Compared with the respective densities in the vehicle group, the new vessel densities of the ischemic penumbra hippocampus (**a**) in rats of the HPX group were markedly increased at 7 days after focal cerebral I/R injury. When the inhibitor of HO-1, ZnPPIX, was given along with HPX to rats after focal cerebral I/R injury, the positive effect of HPX on enhancing the neovascularization of the ischemic penumbra hippocampus (**a**, **b**) was significantly blocked. However, there was no significant difference in the new vessel density of the ischemic hippocampus in rats in the MCAO and vehicle group at 7 days after focal cerebral I/R injury (**a**, **b**). ^*^P < 0.05 vs. sham group; ^#^*P* < 0.05 vs. vehicle group; ^※^*P* < 0.05 vs. HPX group

Compared with the sham group, the MCAO group exhibited a higher new vessel density in the ischemic penumbra hippocampus areas at 7 days after focal cerebral I/R injury (Sham vs. MCAO: 1.17 ± 0.75 vs. 3.67 ± 1.37; *P* < 0.05, *n* = 6, Fig. 3a and b). The new vessel density of the ischemic penumbra hippocampus area at 7 days after focal cerebral I/R injury witnessed a marked growth in those treated with HPX compared with the new vessel density in those in the MCAO group treated with vehicle (Vehicle vs. HPX: 4.00 ± 1.41 vs. 9.17 ± 2.48; *P* < 0.05, *n* = 6, Fig. 3a and b). When the inhibitor of HO-1, ZnPPIX, was given along with HPX to rats subjected to focal cerebral I/R injury, the new vessel density of the ischemic penumbra hippocampus area at 7 days after focal cerebral I/R dropped substantially compared with the respective densities in the HPX group (HPX vs. HPX + ZnPPIX: 9.17 ± 2.48 vs. 3.33 ± 1.97; *P* < 0.05, *n* = 6, Fig. 3a and b). However, the new vessel density of the ischemic penumbra hippocampus area at 7 day after focal cerebral I/R injury in the vehicle group was not significantly different from that in the MCAO group (*P* > 0.05, *n* = 6, Fig. 3a and b).

### HPX prevented the impairment of the blood-brain barrier (BBB) function caused by focal cerebral I/R injury

**Fig. 4:**
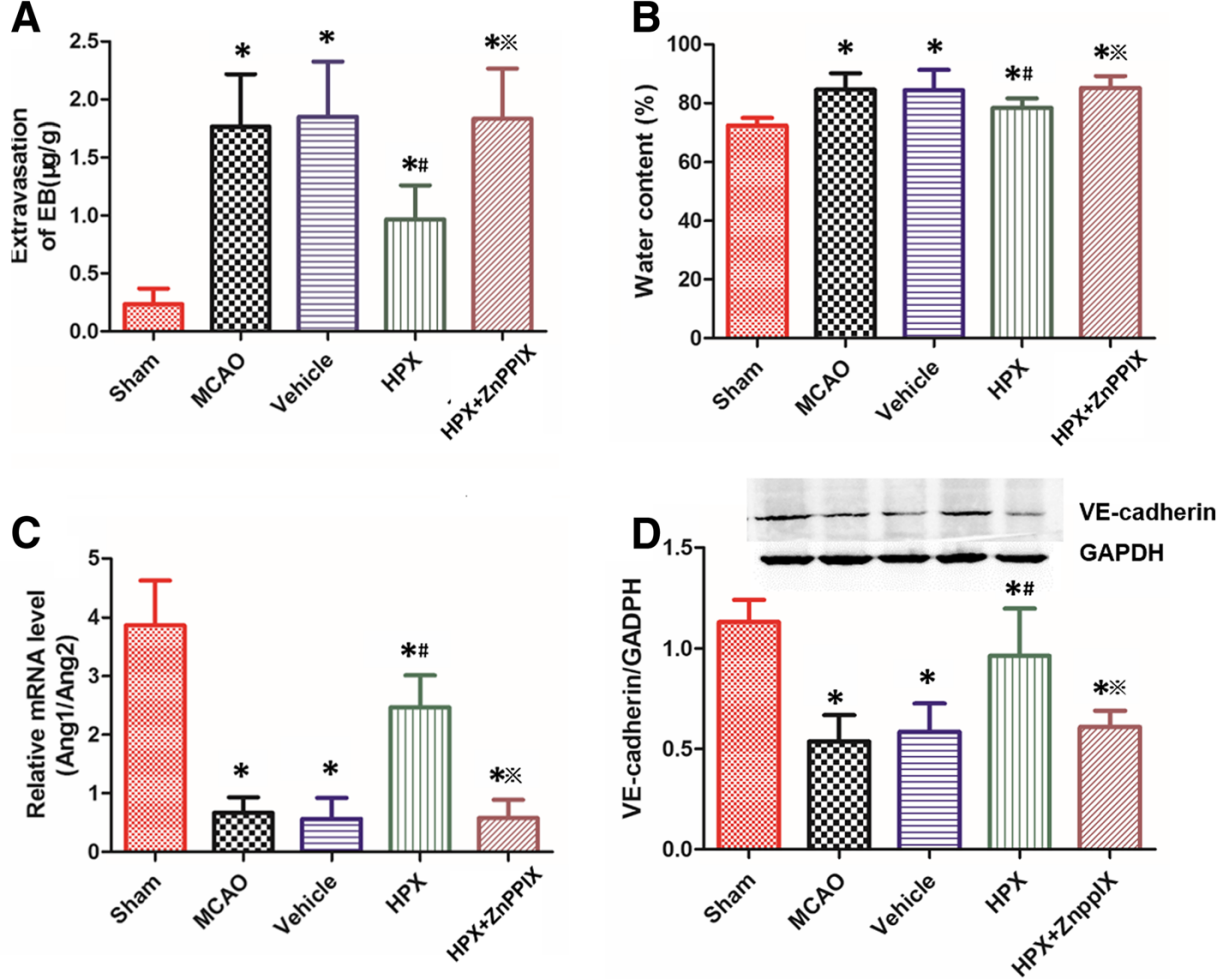
HPX treatment contributed to the repair of the BBB impaired by focal cerebral I/R injury. When compared with the sham operation, the focal cerebral I/R injury resulted in an increase in the extravasation of EB (**a**), brain water content (**b**), along with a decrease in VE-cadherin expression (**d**) and the ratio of the mRNA level of Ang1 and Ang2 (**c**) in rats of the MCAO group. HPX treatment facilitated the repair of the BBB as shown by a reduction in the extravasation of EB (**a**), brain water content (**b**), and the prevention of the decrease in VE-cadherin expression (**d**) and the mRNA ratio of Ang1 to Ang2 (**d**). When ZnPPIX was given along with HPX to rats after I/R injury, the positive effect of HPX on repairing the BBB integrity was blocked (**a, b, c** and **d**). ^*^P < 0.05 vs. sham group; ^#^*P* < 0.05 vs. vehicle group; ^※^*P* < 0.05 vs. HPX group

We evaluated the effect of HPX given via intracerebroventricular injection on alleviating the structural and functional impairment of the BBB by assessing BBB permeability, brain integrity, and new vessel stability at 7 days after focal cerebral I/R injury. Compared with that observed in the sham group, the extravasation of Evans blue dye, which is indicative of the BBB permeability, in the MCAO group increased significantly (Sham vs. MCAO: 0.23 ± 0.14 vs. 1.77 ± 0.45; *P* < 0.05, *n* = 6, Fig. 4a). Furthermore, the extent of brain edema measured by water content, which is indicative of BBB integrity, in rats subjected to focal cerebral I/R injury was more severe than in the sham group (Sham vs. MCAO: 72.33 ± 2.66 vs. 84.50 ± 5.65; *P* < 0.05, *n* = 6, Fig. 4b), and the Ang1/Ang2 ratio, which is associated with the vascular stability, was substantially reduced (Sham vs. MCAO: 3.87 ± 0.76 vs. 0.67 ± 0.27; *P* < 0.05, *n* = 6, Fig. 4c). VE - cadherin plays an important role in endothelial cell migration and survival, angiogenesis and maintaining the integrity of the blood vessels [14]. In our study, VE - cadherin expression decreased after MCAO in contrast to the sham group (Sham vs. MCAO: 1.13 ± 0.11 vs. 0.54 ± 0.13; *P* < 0.05, *n* = 6, Fig. 4d).

When compared with that observed in rats treated with vehicle, the extravasation of Evans blue dye (Vehicle vs. HPX: 1.85 ± 0.48 vs. 0.97 ± 0.29; *P* < 0.05, *n* = 6, Fig. 4a), the water content (Vehicle vs. HPX: 84.33 ± 6.98 vs. 78.33 ± 3.27; *P* < 0.05, *n* = 6, Fig. 4b) and VE - cadherin levels (Vehicle vs. HPX: 0.59 ± 0.14 vs. 0.96 ± 0.23; *P* < 0.05, *n* = 6, Fig. 4d) in HPX group were much higher than vehicle group, and the Ang1/Ang2 ratio was obviously increased (Vehicle vs. HPX: 0.57 ± 0.36 vs. 2.47 ± 0.55; *P* < 0.05, *n* = 6, Fig. 4c) by HPX injection treatment at 7 days after focal cerebral I/R injury. Similarly, these changes were consistent when the MCAO group and HPX group were compared. In rats that received both HPX and ZnPPIX, the antagonist of HO-1, the positive effects of HPX on BBB function were blocked. Specifically, the extravasation of Evans blue dye (HPX + ZnPPIX vs. HPX: 0.97 ± 0.29 vs. 1.83 ± 0.43; *P* < 0.05, *n* = 6, Fig. 4a), the water content (HPX + ZnPPIX vs. HPX: 78.33 ± 3.27 vs. 85.17 ± 4.02; *P* < 0.05, *n* = 6, Fig. 4b) and the VE - cadherin levels (HPX + ZnPPIX vs. HPX: 78.33 ± 3.27 vs. 85.17 ± 4.02; *P* < 0.05, *n* = 6, Fig. 4d) were significantly higher, the Ang1/Ang2 ratio was obviously lower (HPX + ZnPPIX vs. HPX: 0.61 ± 0.0.08 vs. 0.96 ± 0.23, *P* < 0.05, *n* = 6, Fig. 4c) in the HPX + ZnPPIX group than in the HPX group. However, the extravasation of Evans blue dye, the water content, the Ang1/Ang2 ratio and the VE - cadherin protein levels did not differ significantly between the MCAO and vehicle groups (*P* > 0.05, *n* = 6, Fig. 4a-d).

## Discussion

According to the latest study published in *lancet*, stroke has become the first leading cause of death in China and the fourth leading cause of death in the world [15]. Focal cerebral ischemia in stroke patients can lead to severe cognitive impairment or even “vascular dementia”, which would severely affect the life quality of the patients, increase mortality and the recurrence risk of stroke. Recent studies have shown that the damage of the blood-brain barrier and endothelial dysfunction caused by the changes of microvascular environment are the key initiators of neurovascular disorder after cerebral ischemia [13, 16]. Therefore, the endothelial repair and vascular regeneration after cerebral ischemia have become a new intervention target for the recovery of neurological function after stroke.

Hemopexin (HPX) is a plasma protein with the highest affinity to free heme. An increasing number of studies have shown that free heme accumulates in ischemic tissue after I/R, and excessive free heme can be inserted into the vascular endothelium, resulting in damage to the vascular endothelial structural integrity due to its lipophilic properties [11, 17]. Moreover, excessive free heme itself is an inflammatory factor that can induce endothelial and neuronal inflammatory responses and exacerbate endothelial and neuronal damage and dysfunction, resulting in cognitive dysfunction after I/R. Heme-mediated oxidative stress has been revealed to play a fundamental role in various of ischemia-reperfusion pathological processes including ischemia stroke [18]. The HPX-heme complex is transported into cells by endocytosis and then cata-bolismed by heme oxygenase isozymes (HO1 and HO2) [19]. HPX acts to regulate the balance between free heme and bound heme, and/or regulate heme degradation, thus plays a protective role in cell survival, pivotally in short-term cell defense as a powerful heme scavenger. Our previous study has confirmed that the expression of HPX is up-regulated in the ischemic penumbra after I/R in rats, and that intraventricular injection of HPX has a significant neuroprotective effect. However, it remains to be investigated if increasing hemopexin levels in vivo would provide any benefit in improvement of the cognitive dysfunction caused by ischemia-reperfusion injury. In addition, the expression of HO-1 can be induced by the HPX-heme complex formed by the combination of HPX and heme [20]. In an animal model of permanent cerebral infarction, HO-1 has been proved to be benefit on the ultrastructure of the blood-brain barrier [21]. This study aims to explore the effects of HPX on cognitive function after cerebral I/R injury, and to determine the role of HO-1 in the protective effect of HPX during the process.

In this study, therefore, we demonstrated that focal I/R injury prolonged the escape latency in the water maze, decreased the percentage of time in the target quadrant, up-regulated the protein level of HO-1 in the ischemic penumbra and promoted angiogenesis in the hippocampus areas in the MCAO group. Furthermore, injection of HPX in vivo effectively shortened the escape latency in the water maze, increased the percentage of time in the target quadrant, increased the protein expression of HO-1 in the ischemic penumbra and enhanced angiogenesis in the hippocampus areas. These effects were blocked by the HO-1 inhibitor ZnPPIX. Taken together, these data provide novel evidence for the protective role of HPX in improvement of cognitive impairment after cerebral ischemia injury. It was also noteworthy that, HO-1 was pivotal to the process. For us, therefore, it would be critical to pay more attention to the role of HO-1 in HPX treatment of cognitive dysfunction following cerebral I/R. As one of the acute phase proteins, HO-1 plays an endogenous protective role in the body through anti-inflammatory, anti-oxidation and immunomodulation, etc. Besides, HO-1 has been reported to be protective against a variety of vascular-derived lesions by inducing angiogenesis [22]. Furthermore, increased expression of HO-1 in ischemic brain tissue was detected in a cerebral ischemia animal model, among which HO-1 was considered to be protective for the BBB integrity [23, 24]. BBB structure and function damage caused by cerebral ischemia-reperfusion excessive opening of BBB will cause harmful components in blood such as heme to infiltrate into brain tissue thus results in vascular derived brain edema which plays an important role in secondary brain injury. In addition, the exosmosis of blood substances and the destruction of basement membrane during the opening of BBB were beneficial to the accumulation of leukocytes in ischemic brain tissue. The disruption of the micro environment of nerve cells resulted from the inflammatory factors secreted from leukocytes could involve in delayed inflammatory response and aggravate the neurological dysfunction [25].

Hippocampus is the functional brain area of learning and cognition. Its main function is memory formation and short-term memory storage. Previous studies have shown that the hippocampus plays an important role in declarative memory and is responsible for the storage and playback of events [26]. Hippocampus is also involved in non-declarative memory processes such as short-term memory, implicit memory, imagination, perception and cognition. According to our test via immunofluorescence, HPX up-regulated the number of blood vessels in the penumbra hippocampus, and these effects were blocked by the HO-1 inhibitor. It seemed to be rather reasonable, because the neurons and astrocytes in the ischemic penumbra were vulnerable to heme-induced toxicity. More blood vessels in the penumbra hippocampus enabled HPX to arrive at the first time and get rid of overloaded heme generated in ischemia-reperfusion process, thus avoid subsequent irreversible cognitive dysfunction. HPX was also functional in restoration of the BBB disrupted by cerebral I/R as indicated by the reduction in dye extravasations and cerebral edema, enhanced the vascular stability (up-regulated relative mRNA of Ang1/Ang2) and improved endothelial cell tight junction (increased expression of VE- cadherin). HO-1 was also related to the stability of BBB, further strengthens the evidence that HPX improved cognitive dysfunction through HO-1 pathway. However, there still exists several challenges to be conquered, low concentration of HPX in cerebrospinal fluid would be insufficient to cope with the quantities of heme released during ischemia reperfusion. Besides, the mechanisms regulated by HPX in stroke progress are still not fully elucidated, different conclusions might be drawn through different experimental procedure. Fully understanding of the potent mechanisms will provide theoretical foundation to develop the most optimal therapeutic strategies for clinical practice.

In summary, the ability of HPX to up-regulate HO-1 signaling and repair the vascular micro-environment may play an important role in the improved functional outcome observed in rats following ischemia-reperfusion injury.

## Conclusions

HPX can alleviate cognitive dysfunction after focal cerebral ischemia-reperfusion injury through HO-1 pathway and preventing the impairment of the blood-brain barrier in rats.

## Abbreviations

BBB: Blood-brain barrier
CCA: Common carotid artery
EB: Evans Blue
ECA: External carotid artery
EPCs: Endothelial progenitor cells
HO-1: Oxygenase − 1
HPX: Hemopexin
I/R: Ischemia-reperfusion
ICA: Internal carotid artery
MCAO: Middle cerebral artery occlusion
MWM: Morris water maze
RT-PCR: Real-time polymerase chain reaction
vWF: Von Willebrand factor
ZnPPIX: Protoporphyrin IX
